# Functional Dependency Analysis Identifies Potential Druggable Targets in Acute Myeloid Leukemia

**DOI:** 10.3390/cancers12123710

**Published:** 2020-12-10

**Authors:** Yujia Zhou, Gregory P. Takacs, Jatinder K. Lamba, Christopher Vulpe, Christopher R. Cogle

**Affiliations:** 1 Division of Hematology and Oncology, Department of Medicine, College of Medicine, University of Florida, Gainesville, FL 32610-0278, USA; yzhou1996@ufl.edu (Y.Z.); gtakacs@ufl.edu (G.P.T.); 2 Department of Pharmacotherapy and Translational Research, College of Pharmacy, University of Florida, Gainesville, FL 32610-0278, USA; JLamba@cop.ufl.edu; 3 Department of Physiological Sciences, College of Veterinary Medicine, University of Florida, Gainesville, FL 32610-0278, USA; cvulpe@ufl.edu

**Keywords:** acute myeloid leukemia, CRISPR, screening, target identification

## Abstract

**Simple Summary:**

New drugs are needed for treating acute myeloid leukemia (AML). We analyzed data from genome-edited leukemia cells to identify druggable targets. These targets were necessary for AML cell survival and had favorable binding sites for drug development. Two lists of genes are provided for target validation, drug discovery, and drug development. The deKO list contains gene-targets with existing compounds in development. The disKO list contains gene-targets without existing compounds yet and represent novel targets for drug discovery.

**Abstract:**

Refractory disease is a major challenge in treating patients with acute myeloid leukemia (AML). Whereas the armamentarium has expanded in the past few years for treating AML, long-term survival outcomes have yet to be proven. To further expand the arsenal for treating AML, we searched for druggable gene targets in AML by analyzing screening data from a lentiviral-based genome-wide pooled CRISPR-Cas9 library and gene knockout (KO) dependency scores in 15 AML cell lines (HEL, MV411, OCIAML2, THP1, NOMO1, EOL1, KASUMI1, NB4, OCIAML3, MOLM13, TF1, U937, F36P, AML193, P31FUJ). Ninety-four gene KOs met the criteria of (A) specifically essential to AML cell survival, (B) non-essential in non-AML cells, and (C) druggable according to three-dimensional (3D) modeling or ligand-based druggability scoring. Forty-four of 94 gene-KOs (47%) had an already-approved drug match and comprised a drug development list termed “deKO.” Fifty of 94 gene-KOs (53%) had no drug in development and comprised a drug discovery list termed “disKO.” STRING analysis and gene ontology categorization of the disKO targets preferentially cluster in the metabolic processes of UMP biosynthesis, IMP biosynthesis, dihydrofolate metabolism, pyrimidine nucleobase biosynthesis, vitellogenesis, and regulation of T cell differentiation and hematopoiesis. Results from this study serve as a testable compendium of AML drug targets that, after validation, may be translated into new therapeutics.

## 1. Introduction

Acute myeloid leukemia (AML) is challenging to treat due to its refractory nature. Despite achieving initial morphologic remissions in 50–70% of patients, the 10-year disease free survival rate is 16.6% in younger AML patients (<60 years old) and 2.4% in older AML patients (≥60 years old) [1]. Between 2017 and 2019, eight new therapeutics were approved by the US FDA [2]; however, most of these new drugs were developed to target small subgroups of AML based on gene mutations and their approvals were based on short-term clinical improvements such as morphologic remissions and long-term outcomes have yet to be determined. Given the relapsing nature of AML, new therapeutic strategies are needed.

Although AML can be subdivided into subgroups defined by cytogenetic abnormalities and/or genetic mutations, we hypothesized that AML in aggregate depend on a common core of functions. For example, in the clinic we already use pyrimidine analogs as backbone drug agents for the treatment of all AML patients, based on all AML’s essential need for DNA synthesis. We speculated that there may be additional common functions in AML that have yet to be fully investigated. However, searching for leukemia-essential functions has been challenging. In contrast, the scientific strategy of biological reductionism—for example, focusing on FLT3 mutant AML—is generally easier to understand and requires fewer resources than investigating for common essential functions. Major challenges in finding common AML functions is lack of a variety of specimens and culling leukemia-essential from cell-essential. Long-term, inhibiting leukemia-essential functions may be applicable to a greater number of AML patients compared to targeted therapies.

Loss-of-function screens in well annotated cell lines represent a feasible experimental approach to interrogate gene function and cancer cell dependency [3]. The Broad Cancer Dependency Map Consortium (DepMap) is the most comprehensive lentiviral-based genome-wide pooled CRISPR-Cas9 screen designed for this purpose, including screens of 17,634 genes across 1714 cancer cell lines in the Avana 19Q2 library [4]. Additionally, CanSAR Black, the largest database for cancer drug discovery, contains 215,178 experimentally derived protein–ligand interactions and 111,414 three-dimensional (3D) molecular models which may be used to assess possible drug-gene interactions [5]. The combination of identified essential genes and prospective drug-gene interactions may then be compared to characteristics of existing therapeutic targets using the Drug Gene Interaction Database (DGIdb), which annotates known drug-gene interactions [6].

**Figure 1:**
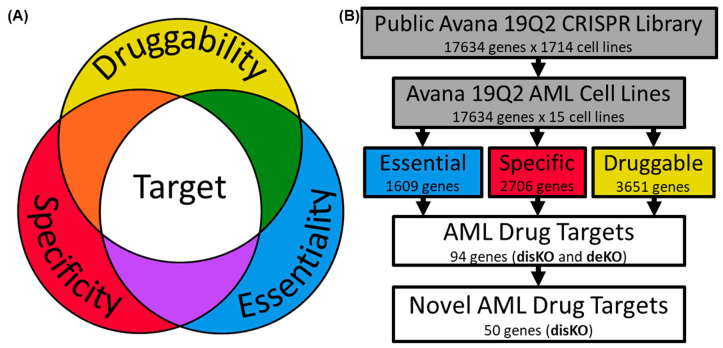
Experimental pipeline for identifying essential and druggable genes in acute myeloid leukemia. Color scheme for gene grouping is consistent throughout panels. (**A**) Venn diagram modelling the ideal characteristics for a safe and effective drug target. (**B**) Public Avana 19Q2 CRISPR Library was curated from the Broad Cancer Dependency Map Consortium (DepMap) consortium to identify gene dependencies in acute myeloid leukemia cell lines. Essentiality, specificity, and druggability thresholds were set at a high specificity (0.90) by receiver–operator curve (ROC) analysis. Genes not targeted by existing approved or experimental drugs are curated in disKO for drug discovery.

We hypothesized that a safe and effective drug target in AML is necessary for AML cell survival (essential), non-essential to non-AML cells (specific), and can be inhibited by drugs (druggable) (Figure 1A). Thus, we designed computational screening methodology combining the DepMap, canSAR Black, and DGIdb databases to identify novel druggable targets for the treatment of AML. Gene ontology and protein–protein interaction analysis were also conducted on the druggable target list to identify biological processes essential for AML cell survival.

## 2. Methods

### 2.1. Examination of AML Dependencies

The DepMap Avana 19Q2 CRISPR screening library contained CRISPR-Cas9 dropout data for 17,634 genes in 15 AML cell lines (HEL, MV411, OCIAML2, THP1, NOMO1, EOL1, KASUMI1, NB4, OCIAML3, MOLM13, TF1, U937, F36P, AML193, P31FUJ) within a library of 1714 cell lines [3]. DepMap calculates dependency scores as the probability that a gene is a member of the distribution of genes essential for cell survival versus a member of the genes composing the non-essential distribution for a given cell line. Dependency scores are specific to each cell line and its respective genome. Scores are additionally processed using CERES, a computational method accounting for copy-number-specific effects and variable sgRNA activity, to reduce false positives [7]. DepMap identified common essential genes, hypothesized to be essential for all human cell viability [8], as the top depleting genes in 90% of cell lines [4]. AML specific data were downloaded and tabulated in IBM SPSS Statistics for Windows (IBM Corp., Armonk, NY, USA). Descriptive statistics were calculated for measuring gene dependency across all AML cell lines. Mean AML dependency was calculated as the average of a gene’s dependency values in AML cell lines. Maximum AML dependency was defined as the gene’s greatest dependency value from any AML cell line. We also calculated AML-specific Z-scores for each gene as the standardized difference between mean AML dependency in 15 AML cell lines and mean dependency in all cell lines in Avana 19Q2 data set.

### 2.2. Assessing AML Gene Druggability

CanSAR Black was queried on 20 June 2020 to obtain druggability measures for each gene product identified above, drawing from experimentally derived ligand-based drug interactions and X-ray crystallography derived computational 3D protein-drug binding in the Protein Data Bank (PDB) [9]. Unmatched queries (*n* = 1585, 8.988%) were assigned null scores and excluded from screening. 3D measurements included number of successful binding models and percentage of models successful at binding for both “druggability” and “tractability”, qualities defined by CanSar but are herein discussed synonymously as druggability. Ligand-based measurements included standardized scores and percentiles, which are directly related. As a result, they were not compared against each other and only the standardized ligand-based score was used.

### 2.3. Determining AML Drug Target Screening Characteristics

Utilizing the Drug Gene Interaction Database (DGIdb) accessed on 20 June 2020, we constructed a list of all known and predicted drug-gene interactions for the genes in Avana 19Q2 [6]. This list was further parsed into those gene products inhibited (i.e., inhibitors, inverse agonists, antagonists, negative modulators, binders, and blockers) by drug therapy. We further divided these druggable gene products into US FDA-approved AML drug therapy, experimental or US FDA-approved drug therapy for all cancers, and US FDA-approved drug therapy for all diseases. Possible measures for ranking drug targets by essentiality (mean vs. max dependency), specificity (common essential genes vs. AML-specific Z-score), and druggability (CanSAR druggability vs. tractability, raw number vs. percentage) were compared in IBM SPSS Statistics software with appropriate parametric and nonparametric tests.

### 2.4. Screening Genes for AML Drug Development

Cutoff values for criteria measurements of essentiality, specificity, and druggability, discussed in results, were set at an estimated specificity of 0.90 based on receiver–operator curve (ROC) analysis of the three drug categories in IBM SPSS. The genes meeting both criteria (i.e., AML specificity and druggable) but not currently targeted by a US FDA-approved or experimental drug therapy formed a drug discovery knock-out list termed “AML disKO.” Genes targeted by existing drugs formed a drug development knockout list termed “AML deKO”. An analysis of functional interactions was conducted using StringDB [10], on the top 94 druggable AML-specific essential genes (including disKO and deKO lists). Minimum required interaction scores were set at high confidence (0.700) and utilized experiments, databases, co-expression, neighborhood, gene fusion, co-occurrence, and text mining to identify interactions. Network display was simplified by hiding disconnected nodes. Markov cluster algorithm (MCL) network clustering (inflation parameter: 3) was applied to the analysis. Gene ontology analysis in StringDB and PANTHER [11] was also conducted on the top 94 druggable AML-specific essential genes to identify enriched biological functions. PANTHER and StringDB were accessed on 24 August 2020.

## 3. Results

### 3.1. Identifying AML-Specific Essential Gene Characteristics

**Figure 2:**
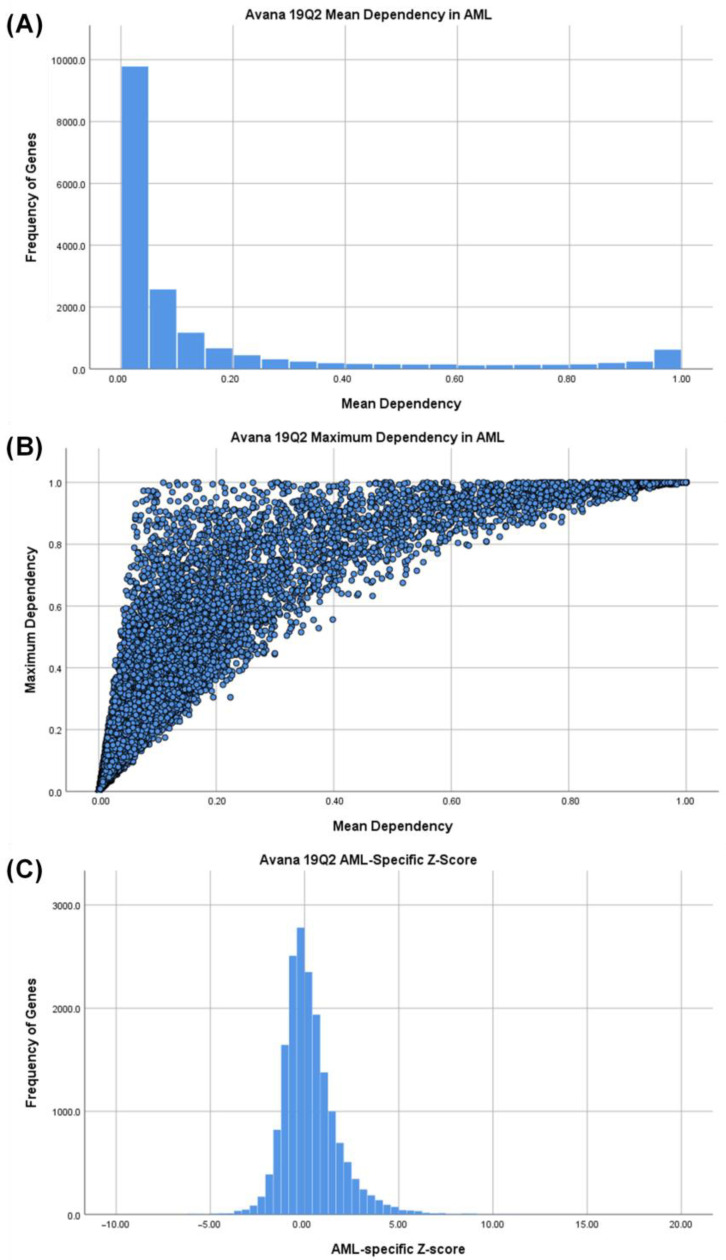
Mean and maximum gene dependency scores from Avana 19Q2 genes and acute myeloid leukemia (AML) cell lines. (**A**) Histogram of dependency scores averaged from 15 AML cell lines from Avana 19Q2 CRISPR Library. N = 17,634, Median = 0.04, Interquartile range = 0.01–0.14. Modes at 1.0 (essential) and 0.0 (non-essential) represent the ideal distributions of genes that AML cells depend on or do not depend on, respectively, for cell growth and survival. (**B**) Data points (N = 17,634) represent genes across 15 AML cell lines from Avana 19Q2. Maximum dependency scores for each gene are on the *y*-axis and mean dependency scores are on the *x*-axis. Maximum dependency may exceed mean dependency at all mean dependency values except 0.0 and 1.0. (**C**) Histogram of AML-specific Z-scores for each gene, calculated as the standardized difference between mean dependency in 15 AML cell lines and mean dependency in all cell lines in Avana 19Q2. The distribution is approximately normal and positively skewed. The positive tail represents a population of genes which may be more essential to survival of AML cells than to other cancer cells.

A stepwise method was used to identify essential, specific, and druggable genes for the treatment of AML (Figure 1B). First, gene dependency scores from Avana 19Q2 data set for 15 AML cell lines were obtained. We noted that mean dependency scores from all 15 AML cell lines followed a bimodal distribution reflecting a division between essential and non-essential genes in AML with the majority of genes showing low mean dependency scores (<0.2) and a smaller subset showing high dependency (>0.95) in AML cells (Figure 2A). However, this distinction inflates the importance of the mean dependency score because some genes with a low mean AML dependency scores have a high maximum AML dependency score (upper left quadrant of Figure 2B), likely reflecting the genetic heterogeneity of the AML cell lines tested. Whereas, the mean score and maximum score are informative, neither one alone can be used to identify AML-specific essential genes.

**Figure 3:**
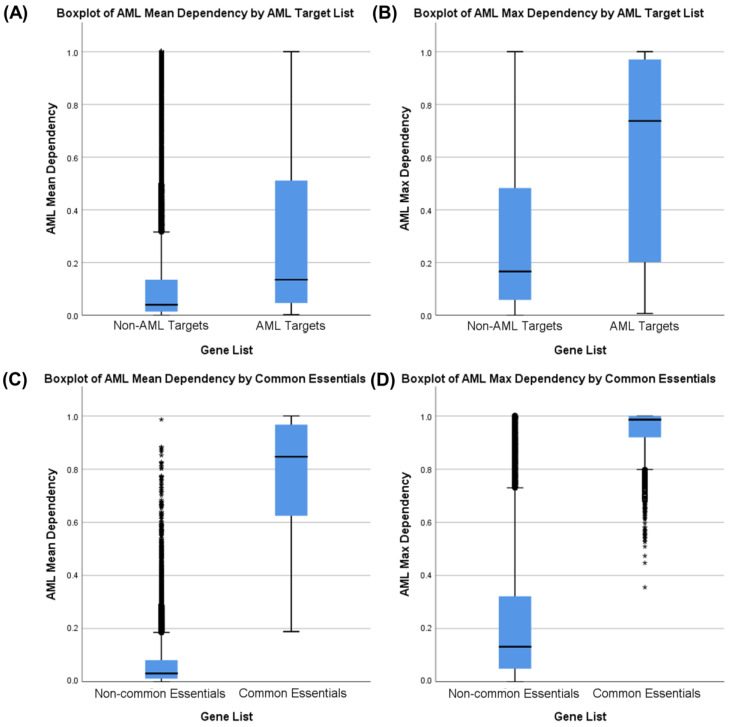
Boxplots and Mann–Whitney tests comparing AML cell line mean dependency and maximum dependency measurements of known AML drug targets and common essential genes. Outliers are displayed as circles (1.5*IQR) or asterisks (3*IQR). Maximum dependency is the best discriminator between AML targets and other targets as measured by U-statistic. (**A**) Boxplot of AML mean dependency in non-AML Avana 19Q2 targets versus AML drug targets. ΔMed. = 0.0952, U = 673,434, two-tailed *p* < 0.001. (**B**) Boxplot of AML max. dependency in non-AML Avana 19Q2 targets versus AML drug targets. ΔMed. = 0.5714, U = 691,368, two-tailed *p* < 0.001. (**C**) Boxplot of AML mean dependency in non-common essential Avana 19Q2 genes versus common essential genes. ΔMed. = 0.8154, U = 32,825,327, two-tailed *p* < 0.001. (**D**) Boxplot of AML max. dependency in non-common essential Avana 19Q2 genes versus common essential genes. ΔMed. = 0.8546, U = 14,618,798, two-tailed *p* < 0.001.

Next, we examined the AML dependency scores of a set of gene products currently targeted by US FDA-approved AML drug therapy. Non-parametric Mann–Whitney U-tests comparing both the mean and maximum AML dependency scores were significantly greater (*p* < 0.001) for these existing leukemia chemotherapeutic drug targets (AML Targets) as compared to a set of all other genes from the Avana 19Q2 dataset (Non-AML Targets) (Figure 3A,B), with a greater difference in maximum AML dependency. In contrast, similar examination of gene products targeted by all antineoplastic therapy or by other FDA-approved drugs did not identify a significant difference (Tables S1–S8). These results suggest that existing AML drugs preferentially inhibit genes identified as essential to AML in DepMap, whereas other drug therapies do not. However, the set of genes inhibited by existing AML drugs could represent either common essential genes (required in all cell lines) or AML-specific essential genes. In fact, non-parametric Mann–Whitney U-tests comparing mean and maximum AML dependency scores in gene lists were significant (*p* < 0.001) for common essential genes (Figure 3C,D), with a greater difference in mean dependency. This could reflect the role of high mean dependency scores for identifying common essential genes, but it also suggests that inhibition of common essential genes is a common mechanism of action in AML drug therapies. Indeed, our results show that AML drug targets with currently approved therapeutics were associated with inhibition of common essential genes while cancer drug targets and other-disease drug targets with approved therapeutics were not associated with inhibition of common essential genes (Table 1 and Tables S9a–S11b). Together, our results show that currently approved AML drugs inhibit AML-specific essential genes but also target common essential genes required in many different cell types. This finding fits with the clinical experience of AML drug therapies eliciting systemic toxicities. On a technical level, these results also indicate that maximum AML dependency is a superior discriminator for identifying AML drug targets compared to mean AML dependency, which is more likely associated with common essential genes. Furthermore, high dependency scores in AML cell lines alone is an insufficient discriminator between AML-specific drug targets and common essential genes.

### 3.2. Identifying AML-Specific Essential Gene Characteristics

To identify AML-specific drug targets with reduced systemic effects, we developed a novel metric, the AML Z-score. The AML Z-score for each gene represents the standardized difference between mean dependency in 15 AML cell lines and mean dependency of all cell lines in the Avana 19Q2 data set. AML-specific Z-score distributions were approximately normal and positive-skewed (Figure 2C). The positive tail indicates genes that may be specifically essential for AML cell survival than other cell types.

**Figure 4:**
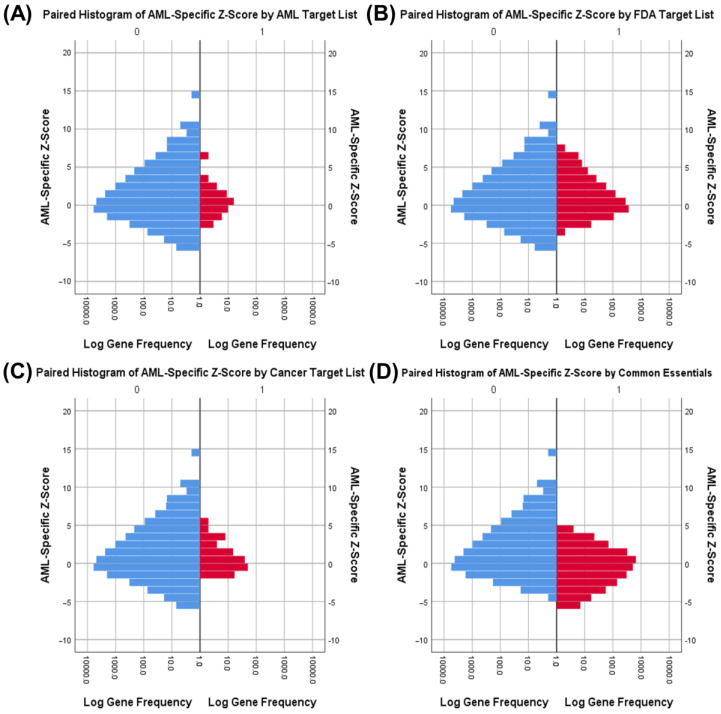
Paired histograms and independent *t*-tests comparing AML-specific Z-scores of AML drug targets (red), FDA drug targets (red), cancer drug targets (red), and common essential genes (red) with all Avana 19Q2 genes (blue). (**A**) Paired histogram of AML-specific Z-scores in non-AML Avana 19Q2 targets versus AML drug targets. ΔMean Z = 0.4645, *df* = 17632, *t* = 2.355, two-tailed *p* = 0.019. If equal variances are not assumed, *p* = 0.059. (**B**) Paired histogram of AML-specific Z-scores in non-FDA Avana 19Q2 targets versus FDA approved drug targets. ΔMean Z = 0.0670, *df* = 17632, *t* = 1.407, two-tailed *p* = 0.159. (**C**) Paired histogram of AML-specific Z-scores in non-cancer Avana 19Q2 targets versus cancer drug targets. ΔMean Z = 0.0553, *df* = 17632, *t* = 0.443, two-tailed *p* = 0.658. (**D**) Paired histogram of AML-specific Z-scores in non-common essential Avana 19Q2 genes versus common essential genes. ΔMean Z = −0.4900, *df* = 17632, *t* = −14.456, two-tailed *p* < 0.001.

Independent samples *t*-tests comparing AML-specific Z-scores were significant (*p* < 0.01) only for genes targeted by common essential genes and AML chemotherapy but not for FDA drug targets or other chemotherapy targets (Figure 4). This indicates that a high AML-specific Z-score is selective for AML drug targets, selective against common essential genes, and not selective for other drug therapies. Common essential genes have significantly lower Z-scores among AML cell lines (Figure 4D), demonstrating that AML cells have genetic dependencies distinct from other human cells which may be exploited to produce AML-specific cell death. We concluded that the combination of high maximum AML dependency and high AML-specific Z-score therefore is a superior discriminator for identifying AML-specific drug targets.

### 3.3. Druggability as a Discriminator

**Figure 5:**
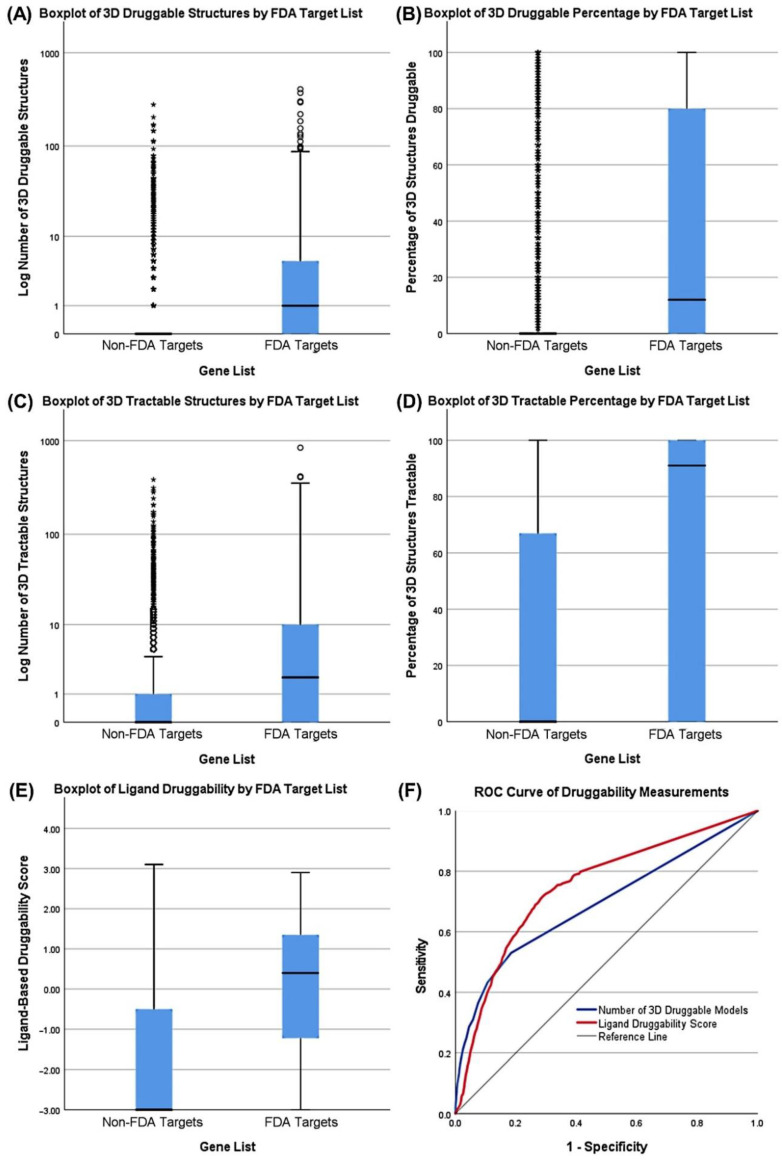
Boxplots, Mann–Whitney tests, and ROC analysis comparing druggability measurements for discriminating FDA drug targets. Outliers are displayed as circles (1.5*IQR) or asterisks (3*IQR). 3D Tractable structures is the best discriminator of FDA targets and other targets as measured by U-statistic (among 3D measurements). (**A**) Boxplot of log number of 3D druggable structures associated with non-FDA Avana 19Q2 targets versus FDA drug targets. ΔMed. = 1, U = 10,520,448, two-tailed *p* < 0.001. (**B**) Boxplot of 3D structures druggable percentage associated with non-FDA Avana 19Q2 targets versus FDA drug targets. ΔMed. = 12%, U = 10,211,976, two-tailed *p* < 0.001. (**C**) Boxplot of log number of 3D tractable structures associated with non-FDA Avana 19Q2 targets versus FDA drug targets. ΔMed. = 2, U = 10,782,284, two-tailed *p* < 0.001. (**D**) Boxplot of log number of 3D structures tractable percentage associated with non-FDA Avana 19Q2 targets versus FDA drug targets. ΔMed. = 91%, U = 10,221,548, two-tailed *p* < 0.001. (**E**) Boxplot of ligand-based druggability score associated with non-FDA Avana 19Q2 targets versus FDA drug targets. ΔMed. = 3.40, U = 11,390,680, two-tailed *p* < 0.001. (**F**) ROC curve of 3D and ligand druggability measurements for predicting FDA approved drug targets. 3D Tractable AUC = 0.709, 95% CI = (0.690, 0.728). Ligand Score AUC = 0.749, 95% CI = (0.733, 0.766).

We used the CanSAR Black database to evaluate the set of candidate AML targets for drug development feasibility. The term “tractable” within CanSAR refers to high predicted efficacy of drug-protein interactions whereas “druggable” also includes an assessment of safety based on potential off-target interactions with related protein families [12]. For simplicity, we refer to both qualities synonymously as druggability. Not surprisingly, all druggability measurements for gene products targeted by FDA-approved drugs were significant (*p* < 0.001, non-parametric Mann–Whitney U-test) compared to other gene products (Figure 5), with the number of 3D tractable structures as the most discriminatory measure of druggability among 3D structure metrics. 3D and ligand measurements from CanSAR Black are distinct methods for assessing druggability and yield different results which do not always agree but serve similar purposes with comparable performance (Figure 5F). Ligand-based measurements are experimentally derived but use a limited library of ligands and proteins. 3D modelling is determined from a variety of flexible computational models but may not translate to experimental results. As a result, a high score in either measurement method was used to screen for druggable genes.

### 3.4. Essential Gene List for AML Drug Discovery

ROC analysis determined the cutoff values and estimated sensitivity correlating with a 0.90 specificity for maximum dependency, AML-specific Z-score, and druggability (Table 2) estimated from respective reference gene lists. From 16,049 genes with available dependency and druggability data, 94 genes (0.6%) met all cutoff values and were judged as good targets for AML pharmacotherapy.

Fifty of these 94 gene products (53%) were not targeted by an existing or experimental drug and were annotated in a list called AML discovery gene knockout set (AML disKO) (Table 3) which represented candidate targets for drug discovery. The remaining 44 of 94 gene products (47%) were targeted by existing or experimental drugs for any disease and annotated in a separate list called AML developmental gene knockout set (AML deKO) (Table 4), representing targets for validation and/or drug repurposing.

We note that while all gene products in disKO and deKO had a high Z-score indicating greater mean dependency in AML cells than other cells, 56/94 (60%) are previously identified common essential genes (red) while 38/94 (40%) are not common essential genes (blue). Targeting of the disKO common essential genes (Table 3, red) may be a viable option but would be expected to have some systemic toxicity; however, less than other common essential genes in non-AML cells. The disKO targets which are not common essential genes (Table 3, blue) represent candidate genes in which inhibition would be expected to have AML-specific effects with limited non-specific effects. The deKO genes, which met identical screening criteria for AML drug characteristics, represent similarly promising targets for repurposing existing drugs for AML.

### 3.5. Essential Physiologic Processes in AML

**Figure 6:**
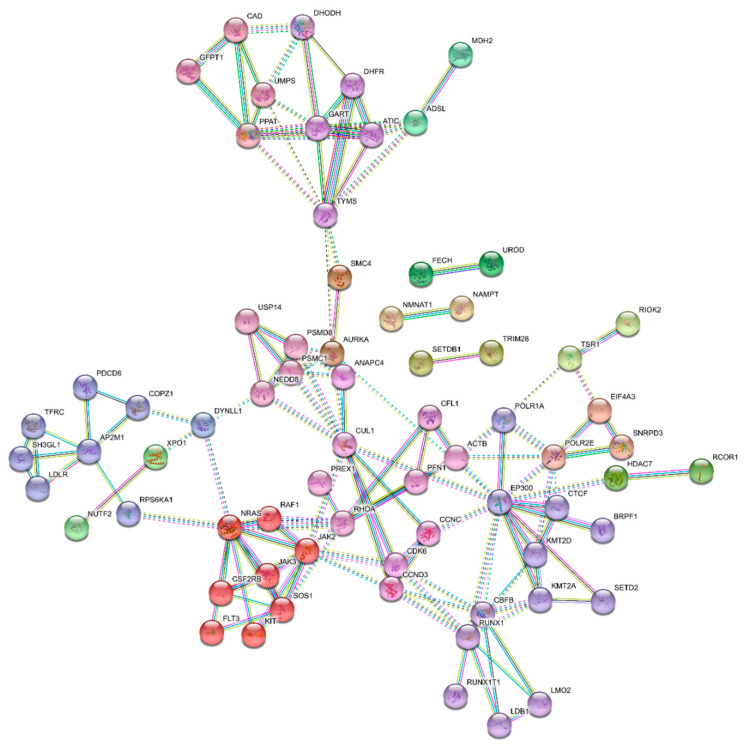
STRING: Protein–protein interaction network functional enrichment analysis among the top 94 identified AML drug targets. Nodes represent proteins. Edges connecting nodes represent protein–protein associations. Associations are based on evidence and require a high confidence (0.700) minimum required interaction score. Display was simplified by hiding disconnected nodes in the network. MCL network clustering (inflammation parameter: 3) was applied. Associations do not necessarily mean that the proteins are physically bound. Interaction types: Curated databases (turquoise), experimentally determined (magenta), gene neighborhood (green), gene fusions (red), gene co-occurrence (blue), co-expression (black), protein homology (purple), text mining (yellow). Dashed lines represent strongest interaction scores. Number of nodes: 94; Number of edges: 144; Average node degree: 3.06; Avg. local clustering coefficient: 0.509; Expected number of edges: 86; PPI enrichment *p*-value: 5.47 × 10^−9^.

STRING analysis identified clustering around distinct protein interaction clusters (Figure 6). Furthermore, this network of the top 94 druggable AML-specific essential genes had significantly more interactions among themselves than would be expected for a set of proteins of similar size, randomly selected from the human genome. Such an enrichment indicates that the proteins are functionally connected as a group.

**Figure 7:**
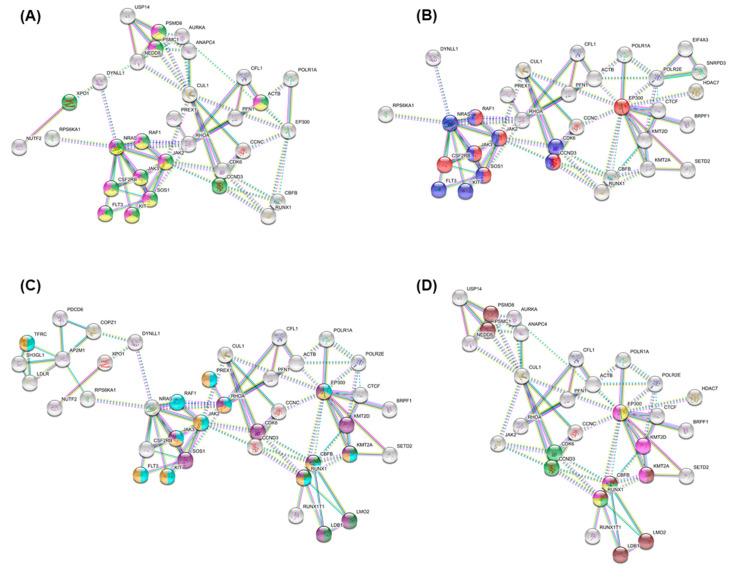
STRING: AML associated enriched pathways among the top 94 identified drug targets. All settings are consistent with Figure 6. Highlighted proteins represent functional enrichment. Display was simplified by hiding non-enriched proteins. Panels represent different enriched pathways. (**A**) MAPK signaling: MAPK family signaling cascades (green), MAPK1/MAPK3 signaling (yellow), RAF/MAP kinase cascade (pink). (**B**) JAK-STAT/PI3K-AKT signaling pathway: JAK-STAT signaling pathway (red), PI3K-AKT signaling pathway (blue). (**C**) Hematopoiesis, stem cell, and hemopoiesis: Regulation of hematopoietic stem cell differentiation (green), hematopoietic or lymphoid organ development (blue), hemopoiesis (yellow), regulation of hemopoiesis (purple). (**D**) RUNX1 related pathways: RUNX1 regulates transcription of genes involved in differentiation of HSCs (brown), Regulation of RUNX1 expression and activity (green), RUNX3 regulates p14-ARF (yellow), RUNX1 regulates genes involved in megakaryocyte differentiation and platelet function (pink).

Pathway analysis conducted through STRING highlighted proteins among our top AML essential genes, with enrichment in MAPK signaling (Figure 7A), JAK-STAT/PI3K-AKT signaling pathway (Figure 7B), hematopoiesis, stem cell, and hemopoiesis (Figure 7C), and RUNX1 related pathways (Figure 7D). PANTHER gene ontology analysis additionally identified top enriched biological processes involving de novo uridine monophosphate (UMP) biosynthetic process, de novo inosine 5′-monophosphate (IMP) biosynthetic process, dihydrofolate metabolic process, de novo pyrimidine nucleobase biosynthetic process, vitellogenesis, and positive regulation of CD8-positive/alpha-beta T cell differentiation (Table 5). Together, these results show AML cell vulnerability within metabolic processing and suggest that these enriched pathways are required for leukemia cell survival.

## 4. Discussion

In this study we generated a list of druggable gene targets specific to AML through the examination of large datasets of genome-wide pooled CRISPR screening and pharmacotherapy modeling. Our approach contrasts with recent efforts that focus on AML subgroups defined by genomic mutation. For example, a recent study used only five AML cell lines to identify candidate AML drug targets, three of which (60%) contained *MLL* translocations, which likely skewed their results toward *MLL*-driven AML [13]. The prior CRISPR screen identified five gene KOs leading to AML dropout in all five cell lines, including *AURKB*, *MAP2K1*, *MAP2K2*, *IGF1R*, and *HDAC3*. Although none of these targets passed our screening criteria, related genes such as *AURKA*, *HDAC7*, *RUNX1*, and *KMT2A* were identified as targets with existing inhibitors (Table 4). We note that inhibitors to AURKB and *MAP2K* have already shown poor efficacy in AML clinical trials although it may be worthwhile to explore these targets specially in *MLL*-translocated AML [14,15]. In this study we aggregate a large number of AML sources (15 AML cell lines) with the intent to find common functions among all AML subtypes. This strategy admittedly comes at the sacrifice of finding mutation-specific vulnerabilities (such as SYK in FL3-mutant AML), but at the potential benefit of finding shared targets applicable to a greater number of patients.

The genes identified in our deKO list (Table 4) are targets of already approved drugs, which provides an internal validation of our computational method. In particular, the genes FLT3 and KIT from our deKO list are known targets in AML. The FLT3/KIT inhibitors Midostaurin [16] and Quizartinib [17], were recently approved for AML treatment in the US and Japan respectively. The hypomethylating agents Azacitidine and Cytarabine, frequently used in AML, were also shown to enhance FLT3 and KIT inhibition in combination with both Midostaurin, Quizartinib [17,18]. MCL1 is a known target in AML with drugs in development, alone and in combination with other anti-apoptosis inhibitors such as venetoclax [19].

Other deKO genes with inhibitors designed for other diseases may also represent drugs for repurposing. We found LDLR as a highly essential gene in AML cell lines, indicating that cholesterol metabolism is essential in AML. Pravastatin in phase 2 Southwest Oncology Group (SWOG) clinical trials has shown improved morphologic remission in combination with Idarubicin and Cytarabine [20].

**Figure 8:**
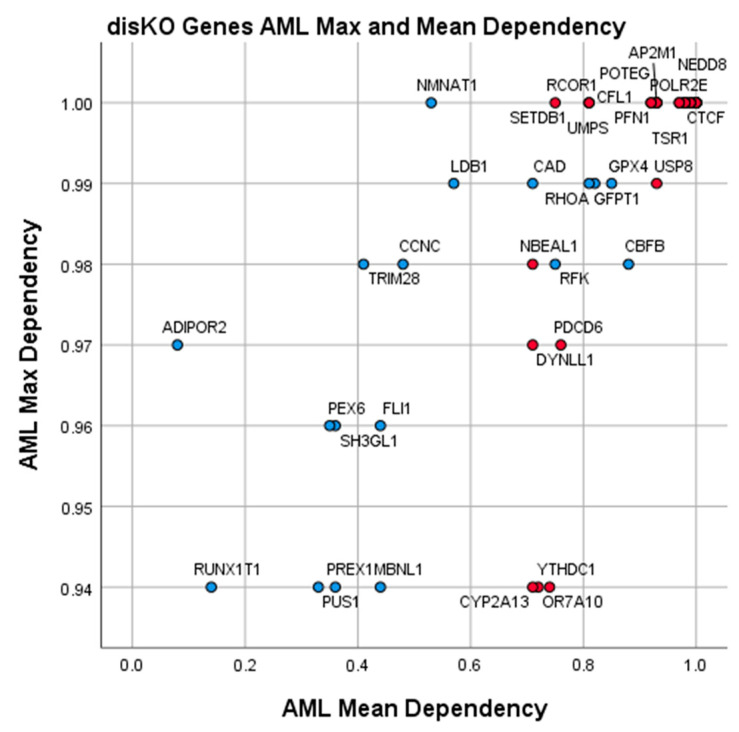
Scatter plot of mean and maximum gene dependency scores of disKO genes in 15 AML cell lines from Avana 19Q2. Data points (*n* = 50) represent genes curated in disKO. Overlapping points may obfuscate some genes. Common essential genes are red and non-common essential genes are blue. Maximum dependency scores for each gene are on the *y*-axis and mean dependency scores are on the *x*-axis.

Additionally, we identified novel targets for AML drug discovery in our disKO list (Table 3). These targets have distinct advantages for drug discovery because common essential gene inhibition is more likely to induce toxicity across many AML genotypes while AML-specific essential gene inhibition is less likely to cause systemic toxicity. A scatterplot of the disKO genes are presented in Figure 8. Gene-KOs shown in red in the upper outer quadrant represent particularly attractive targets given their essentiality and specificity for AML. These AML-specific, non-common essential genes in disKO such as CBFB may have lower mean dependency scores relative to common essential genes, but are highly essential and specific to at least one AML cell line. If Hart et al. is correct that inhibition of common essential genes is more likely to produce clinical toxicity, then inhibiting these AML-specific essential drug targets will likely be safer. AML cases with CBFB translocations are known to be responsive to cytarabine chemotherapy and exhibit favorable clinical prognosis in pediatric and adult patients [21,22]. Experimental studies have shown CBFB as an essential gene in several AML genotypes [23,24]. Mouse models of AML show that inhibition of CBFB has anti-leukemic activity [25]. Our results confirm CBFB as a druggable vulnerability in AML and expand the knowledgebase by providing many other drug targets for focused investigation.

Our results highlight the importance of AML metabolism and opportunities for therapeutic intervention. Uridine and inosine biosynthesis are known essentials for AML cell proliferation and differentiation [26,27]. Recently a dihydrofolate reductase (DHFR) antagonist was identified as a potent and selective inhibitor of AML [28]. It was recently demonstrated that leukemia stem cells were not reliant on amino acid metabolism due to their ability to compensate through increased fatty acid metabolism [29].

When interpreting our results, several considerations need to be made. First, the disKO list was constructed from a genome-wide data analysis. Before embarking on any drug development work, target validation testing is needed. Second, Avana 19Q2 included only 15 cell lines, which may not fully represent the heterogeneity of AML. Follow-up tests using primary AML cells would validate the AML cell-line results in this study and add clinical relevance. Third, in vivo experimental data is also needed before clinical testing.

In conclusion, we used an agnostic, genome-wide approach to identify potential new therapeutic targets for AML and provide hit lists for target validation, drug discovery, and drug development.

## 5. Conclusions

Data analysis of an agnostic, genome-wide CRISPR screen led to the identification of 94 druggable genes specific and essential for AML survival. These genes represent potential drug targets after validation testing.

## Figures and Tables

**Table 1:** Contingency tables and Fisher’s Exact tests for association of common essential genes with AML, cancer, and FDA drug targets. AML drug targets and common essential genes are significantly associated (underlined), while cancer drugs and FDA approved drugs are not significantly associated with common essential genes.

Fisher’s Exact Tests for Common Essential Genes by Gene List				
Target List		Common Essential Genes	Non-Common Essential Genes	Significance (2-Sided)
AML Drug Targets	AML	Actual: 17	Actual: 39	*p* < 0.001
		Expected: 6.7	Expected: 49.3	
	Other	Actual: 2108	Actual: 15,470	
		Expected: 2118.3	Expected: 15,459.7	
Cancer Drug Targets	Cancer	Actual: 18	Actual: 122	*p* = 0.806
		Expected: 16.9	Expected: 123.1	
	Other	Actual: 2107	Actual: 15,387	
		Expected: 2108.1	Expected: 15,385.9	
All FDA Drug Targets	FDA	Actual: 104	Actual: 914	*p* = 0.069
		Expected: 122.7	Expected: 895.3	
	Other	Actual: 2021	Actual: 14,595	
		Expected: 2002.3	Expected: 14,613.7	

**Table 2:** Cutoff values and estimated sensitivity for drug target screening. A specificity of 0.90 was established as a cutoff. Cutoff values and sensitivity were calculated from ROC analysis by linear interpolation of neighboring values if no data point was exactly 0.90. Reference gene lists were used as the standard for determining AML drug target characteristics in ROC analysis.

Characteristic	Reference List	Measurement	Cutoff	Sensitivity
Essentiality	AML Drug Targets	AML Max. Dependency	0.9361	0.309
Specificity	Non-common essential genes	AML-Specific Z-Score	1.4268	0.178
Druggability (3D)	FDA Targets	Number of TracTable 3D Structures	4.0652	0.400
Druggability (Ligand)	FDA Targets	Ligand-Based Score	0.8875	0.382

**Table 3:** AML disKO drug target discovery list. This table contains a list of genes for which (**a**) knock-out led to AML dropout, (**b**) there were no drugs specifically targeting the gene-product, and (**c**) the gene-product was druggable. The genes were ranked by mean dependency in 15 AML cell lines and color coded for non-common essential genes (blue) and common essential genes (red) as identified by DepMap. Gene descriptions are linked to the National Center for Biotechnology Information (NCBI) gene database.

Discovery Knockout Genes (AML disKO)						
Gene	AML Mean Dependence	AML Max. Dependence	AML-Specific Z-Score	3D Tractable Structures	Ligand Score	Gene Description
*CBFB*	0.88	0.98	8.39	5	−3.00	Core-binding factor subunit beta
*GPX4*	0.85	0.99	2.85	6	−3.00	Glutathione peroxidase 4
*RHOA*	0.82	0.99	4.27	39	−1.60	Ras homolog family member A
*UMPS*	0.81	1.00	3.49	33	−1.30	Uridine monophosphate synthetase
*GFPT1*	0.81	0.99	4.73	13	−1.30	Glutamine-F6P transaminase 1
*RFK*	0.75	0.98	3.92	4	−3.00	Riboflavin kinase
*CAD*	0.71	0.99	4.30	36	0.90	Carbamoyl-phosphate synthetase 2, aspar …
*LDB1*	0.57	0.99	6.37	6	−0.90	LIM domain binding 1
*NMNAT1*	0.53	1.00	2.45	5	−3.00	Nicotinamide nucleotide acetyltransferase 1
*CCNC*	0.48	0.98	1.57	29	1.55	Cyclin C
*FLI1*	0.44	0.96	8.83	5	−3.00	Fli-1 proto-oncogene, ETS transcription fa …
*MBNL1*	0.44	0.94	3.26	5	−3.00	Muscleblind like splicing regulator 1
*TRIM28*	0.41	0.98	2.53	5	−3.00	Tripartite motif containing 28
*PREX1*	0.36	0.94	8.55	9	−0.95	PIP3 dependent Rac exchange factor 1
*SH3GL1*	0.36	0.96	3.07	0	1.35	SH3 domain containing GRB2 like 1, endo …
*PEX6*	0.35	0.96	1.44	0	1.00	Peroxisomal biogenesis factor 6
*PUS1*	0.33	0.94	3.78	5	1.25	Pseudouridine synthase 1
*RUNX1T1*	0.14	0.94	3.89	4	−3.00	RUNX1 partner transcriptional corepressor 1
*ADIPOR2*	0.08	0.97	3.85	4	−3.00	Adiponectin receptor 2
*POLR2E*	1.00	1.00	1.61	10	−3.00	RNA polymerase II, I and III subunit E
*TSR1*	1.00	1.00	2.09	5	−3.00	TSR1 ribosome maturation factor
*CTCF*	1.00	1.00	1.43	6	−3.00	CCCTC-binding factor
*NEDD8*	1.00	1.00	1.55	11	−3.00	NEDD8 ubiquitin like modifier
*MIS18A*	1.00	1.00	1.56	0	1.35	MIS18 kinetochore protein A
*ANAPC4*	1.00	1.00	1.60	10	−3.00	Anaphase promoting complex subunit 4
*POLR1A*	1.00	1.00	1.51	0	1.40	RNA polymerase I subunit A
*EIF4A3*	0.99	1.00	1.57	10	−3.00	Eukaryotic translation initiation factor 4A3
*SMC4*	0.99	1.00	1.61	1	0.90	Structural maintenance of chromosomes 4
*NUTF2*	0.99	1.00	1.56	4	−3.00	Nuclear transport factor 2
*TFRC*	0.98	1.00	2.52	13	−3.00	Transferrin receptor
*COPZ1*	0.98	1.00	1.51	11	−3.00	COPI coat complex subunit zeta 1
*ADSL*	0.98	1.00	2.29	4	−3.00	Adenylosuccinate lyase
*TERF2*	0.98	1.00	1.70	11	−3.00	Telomeric repeat binding factor 2
*SNRPD3*	0.98	1.00	1.52	9	0.30	Small nuclear ribonucleoprotein D3 polype …
*CUL1*	0.97	1.00	1.57	8	−0.50	Cullin 1
*RIOK2*	0.97	1.00	1.98	6	1.80	RIO kinase 2
*AP2M1*	0.93	1.00	2.43	13	−0.95	Adaptor related protein complex 2 subunit …
*POTEG*	0.93	1.00	2.33	0	1.00	POTE ankyrin domain family member G
*USP8*	0.93	0.99	1.64	5	−1.85	Ubiquitin specific peptidase 8
*PFN1*	0.93	1.00	1.85	5	−3.00	Profilin 1
*CFL1*	0.92	1.00	3.49	8	−3.00	Cofilin 1
*UROD*	0.92	1.00	3.12	19	−3.00	Uroporphyrinogen decarboxylase
*SETDB1*	0.81	1.00	2.35	15	−3.00	SET domain bifurcated histone lysine met …
*PDCD6*	0.76	0.97	1.62	10	−3.00	Programmed cell death 6
*RCOR1*	0.75	1.00	2.66	9	−0.05	REST corepressor 1
*YTHDC1*	0.74	0.94	2.36	36	−3.00	YTH domain containing 1
*OR7A10*	0.72	0.94	1.96	0	2.75	Olfactory receptor family 7 subfamily A me …
*DYNLL1*	0.71	0.97	1.87	7	−3.00	Dynein light chain LC8-type 1
*CYP2A13*	0.71	0.94	2.65	8	1.15	cytochrome P450 family 2 subfamily A me …
*NBEAL1*	0.71	0.98	2.65	0	0.95	Neurobeachin like 1

**Table 4:** AML deKO existing drug target list. This table contains a list of genes for which (**a**) knock-out led to AML dropout, and (**b**) there were drugs specifically targeting the gene-product. The genes are were ranked by mean dependency in 15 AML cell lines and color coded for non-common essential genes (blue) and common essential genes (red) as identified by DepMap. Known targets in AML are bolded. Gene descriptions are linked to the National Center for Biotechnology Information (NCBI) gene database.

Developing Knockout Genes (AML deKO)						
Gene	AML Mean Dependence	AML Max. Dependence	AML-Specific Z-Score	3D Tractable Structures	Ligand Score	Gene Description
*CDK6*	0.86	1.00	3.30	16	0.20	Cyclin dependent kinase 6
*NAMPT*	0.76	1.00	5.95	63	0.30	Nicotinamide phosphoribosyltransferase
*KMT2D*	0.76	0.99	3.40	1	1.35	Lysine methyltransferase 2D
*CCND3*	0.73	0.99	6.64	1	1.65	Cyclin D3
*EP300*	0.72	1.00	3.19	32	1.05	E1A binding protein p300
*PPAT*	0.71	1.00	2.29	0	1.65	Phosphoribosyl pyrophosphate amidotrans...
*PI4KB*	0.67	0.97	3.86	14	0.35	Phosphatidylinositol 4-kinase beta
*ATIC*	0.62	0.99	2.16	5	−0.40	5-aminoimidazole-4-carboxamide ribonucl …
*FECH*	0.62	0.97	3.60	23	−3.00	Ferrochelatase
*TYMS*	0.59	1.00	1.43	60	1.10	Thymidylate synthetase
*GART*	0.56	0.98	3.72	29	0.05	Phosphoribosylglycinamide formyltransfer …
*BRPF1*	0.53	0.94	2.71	56	1.10	Bromodomain and PHD finger containing 1
*KMT2A*	0.50	0.96	4.01	19	−0.25	Lysine methyltransferase 2A
*LMO2*	0.49	0.99	14.21	5	−3.00	LIM domain only 2
*SOS1*	0.49	0.99	3.23	59	0.05	SOS Ras/Rac guanine nucleotide exchan …
*RAF1*	0.42	1.00	5.47	6	1.00	Raf-1 proto-oncogene, serine/threonine ki …
*RPS6KA1*	0.41	0.94	7.38	6	0.80	Ribosomal protein S6 kinase A1
*MDH2*	0.36	0.99	3.41	7	−0.55	Malate dehydrogenase 2
*LDLR*	0.33	0.99	3.21	11	0.20	Low density lipoprotein receptor
*FLT3*	0.31	0.99	3.05	7	1.65	Fms related receptor tyrosine kinase 3
*NRAS*	0.30	1.00	2.89	4	−0.10	NRAS proto-oncogene, GTPase
*RUNX1*	0.30	1.00	6.77	5	−1.45	RUNX family transcription factor 1
*HDAC7*	0.29	0.99	2.46	5	1.20	Histone deacetylase 7
*USP14*	0.25	0.95	2.52	8	−1.90	Ubiquitin specific peptidase 14
*JAK3*	0.17	1.00	1.74	35	1.60	Janus kinase 3
*ETV6*	0.15	0.99	3.56	4	1.45	ETS variant transcription factor 6
*JAK2*	0.13	0.99	8.95	93	1.90	Janus kinase 2
*KIT*	0.13	0.97	1.44	23	1.50	KIT proto-oncogene, receptor tyrosine kina …
*CSF2RB*	0.12	0.99	5.13	5	−3.00	Colony stimulating factor 2 receptor subun …
*TYRP1*	0.08	0.97	3.50	9	−3.00	Tyrosinase related protein 1
*XPO1*	1.00	1.00	1.48	6	−1.25	Exportin 1
*HSPA5*	0.99	1.00	1.56	26	−0.95	Heat shock protein family A (Hsp70) mem …
*WNK1*	0.99	1.00	2.08	6	−1.90	WNK lysine deficient protein kinase 1
*AURKA*	0.98	0.99	1.51	154	1.60	Aurora kinase A
*AHCY*	0.94	1.00	1.96	8	0.75	Adenosylhomocysteinase
*MCL1*	0.88	1.00	3.61	95	0.15	MCL1 apoptosis regulator, BCL2 family m …
*PSMC1*	0.88	0.97	1.75	25	−3.00	Proteasome 26S subunit, ATPase 1
*FDPS*	0.86	0.99	2.24	88	1.05	Farnesyl diphosphate synthase
*PSMD8*	0.85	0.95	1.73	14	−3.00	Proteasome 26S subunit, non-ATPase 8
*ACTB*	0.82	0.99	1.92	18	−3.00	Actin beta
*DHFR*	0.77	1.00	1.61	79	1.25	Dihydrofolate reductase
*HUS1*	0.73	0.99	1.83	4	−3.00	HUS1 checkpoint clamp component
*DHODH*	0.71	1.00	1.77	71	1.05	Dihydroorotate dehydrogenase
*SETD2*	0.70	0.95	1.69	18	1.20	SET domain containing 2, histone lysine m …

**Table 5:** PANTHER gene ontology biological process enrichment analysis among top identified AML essential genes. Gene ontology analysis was conducted to identify enriched biological processes among the top identified AML essential genes. Fisher’s exact test and FDR correction were applied. Biological processes with a *p* value of <0.001 and enriched over 50% are displayed.

PANTHER Gene Overrepresentation Test						
GO Biological Process Complete	Homo Sapiens REFLIST (20851)	Queried	Expected	Fold Enrichment	*p*-Value	FDR
‘de novo’ UMP biosynthetic process (GO:0044205)	3	3	0.01	>100	2.03E−06	2.81E−04
‘de novo’ IMP biosynthetic process (GO:0006189)	6	4	0.03	>100	9.64E−08	2.85E−05
dihydrofolate metabolic process (GO:0046452)	3	2	0.01	>100	2.19E−04	1.37E−02
‘de novo’ pyrimidine nucleobase biosynthetic process (GO:0006207)	7	4	0.03	>100	1.51E−07	3.71E−05
vitellogenesis (GO:0007296)	4	2	0.02	>100	3.28E−04	1.87E−02
positive regulation of CD8-positive, alpha-beta T cell differentiation (GO:0043378)	4	2	0.02	>100	3.28E−04	1.87E−02
pyrimidine nucleobase biosynthetic process (GO:0019856)	9	4	0.04	93.61	3.25E−07	6.56E−05
tetrahydrofolate biosynthetic process (GO:0046654)	7	3	0.03	90.26	1.20E−05	1.29E−03
IMP biosynthetic process (GO:0006188)	10	4	0.05	84.25	4.53E−07	8.41E−5
myeloid progenitor cell differentiation (GO:0002318)	6	2	0.03	70.21	6.08E−04	3.06E−02
histone H3-K4 dimethylation (GO:0044648)	6	2	0.03	70.21	6.08E−04	3.05E−02
regulation of CD8-positive, alpha-beta T cell differentiation (GO:0043376)	6	2	0.03	70.21	6.08E−04	3.04E−02
folic acid-containing compound biosynthetic process (GO:0009396)	9	3	0.04	70.21	2.18E−05	2.10E−03
nucleobase biosynthetic process (GO:0046112)	19	6	0.09	66.51	1.57E−09	9.30E−07
IMP metabolic process (GO:0046040)	13	4	0.06	64.8	1.07E−06	1.70E−04
UMP metabolic process (GO:0046049)	10	3	0.05	63.18	2.83E−05	2.58E−03
UMP biosynthetic process (GO:0006222)	10	3	0.05	63.18	2.83E−05	2.57E−03
pyrimidine ribonucleoside monophosphate biosynthetic process (GO:0009174)	10	3	0.05	63.18	2.83E−05	2.55E−03
pyrimidine ribonucleoside monophosphate metabolic process (GO:0009173)	10	3	0.05	63.18	2.83E−05	2.54E−03
pyrimidine nucleobase metabolic process (GO:0006206)	17	5	0.08	61.95	5.26E−08	1.65E−05
regulation of ribonucleoprotein complex localization (GO:2000197)	7	2	0.03	60.18	7.79E−04	3.73E−02
brainstem development (GO:0003360)	7	2	0.03	60.18	7.79E−04	3.72E−02
pyrimidine nucleoside monophosphate biosynthetic process (GO:0009130)	15	4	0.07	56.16	1.72E−06	2.55E−04
positive regulation of CD8-positive, alpha-beta T cell activation (GO:2001187)	8	2	0.04	52.65	9.71E−04	4.43E−02
positive regulation of synaptic vesicle endocytosis (GO:1900244)	8	2	0.04	52.65	9.71E−04	4.41E−02
pyrimidine nucleoside monophosphate metabolic process (GO:0009129)	16	4	0.08	52.65	2.14E−06	2.95E−04
histone H3-K4 monomethylation (GO:0097692)	8	2	0.04	52.65	9.71E−04	4.40E−02
ribonucleoside monophosphate biosynthetic process (GO:0009156)	33	8	0.16	51.06	1.57E−11	1.79E−08

## Supplementary Materials

The following are available online at https://www.mdpi.com/2072-6694/12/12/3710/s1, Table S1: U Test of AML_dep_mean across Common_Essential, Table S2: U Test of AML_dep_mean across FDA_inhibitor, Table S3: U Test of AML_dep_mean across Cancer_inhibitor, Table S4: U Test of AML_dep_mean across AML_inhibitor, Table S5: U Test of AML_dep_max across Common_Essential, Table S6: U Test of AML_dep_max across FDA_inhibitor, Table S7: U Test of AML_dep_max across Cancer_inhibitor, Table S8: U Test of AML_dep_max across AML_inhibitor, Table S9a: Contingency table of FDA_inhibitor * Common_Essential, Table S9b: Chi-Squared analysis of FDA_inhibitor * Common_Essential, Table S10a: Contingency table of Cancer_inhibitor * Common_Essential, Table S10b: Chi-Squared analysis of Cancer_inhibitor * Common_Essential, Table S11a: Contingency table of AML_inhibitor * Common_Essential, Table S11b: Chi-Squared analysis of AML_inhibitor * Common_Essential,
