# Supplementary material for: Functional Dependency Analysis Identifies Potential Druggable Targets in Acute Myeloid Leukemia
Source: Cancers (Basel). 2020 Dec 10;12(12):3710. doi: 10.3390/cancers12123710 (PMC7764352; doi:10.3390/cancers12123710)
Supplement: Supplementary file 1 [file cancers-12-03710-s001.pdf]

Supplementary Information

Functional Dependency Analysis Identifies Potential Druggable Targets in Acute Myeloid Leukemia

Yujia Zhou [1,+], Gregory P. Takacs [1,+], Jatinder K. Lamba [2], Christopher Vulpe [3] and Christopher R. Cogle [1*]

Table S1. Independent-Samples Mann-Whitney U Test of AML_dep_mean across Common_Essential

Total N	17634
Mann-Whitney U	32825327.000
Wilcoxon W	35084202.000
Test Statistic	32825327.000
Standard Error	220073.867
Standardized Test Statistic	74.280
Asymptotic Sig.(2-sided test)	.000

Table S2. Independent-Samples Mann-Whitney U Test of AML_dep_mean across FDA_inhibitor

Total N	17634
Mann-Whitney U	8110199.000
Wilcoxon W	8628870.000
Test Statistic	8110199.000
Standard Error	157664.617
Standardized Test Statistic	-2.203
Asymptotic Sig.(2-sided test)	.028

Table S3. Independent-Samples Mann-Whitney U Test of AML_dep_mean across Cancer_inhibitor

Total N	17634
Mann-Whitney U	1228086.000
Wilcoxon W	1237956.000
Test Statistic	1228086.000
Standard Error	59993.706
Standardized Test Statistic	.058
Asymptotic Sig.(2-sided test)	.953

Table S4. Independent-Samples Mann-Whitney U Test of AML_dep_mean across AML_inhibitor

Total N	17634
Mann-Whitney U	673434.000
Wilcoxon W	675030.000
Test Statistic	673434.000
Standard Error	38034.337
Standardized Test Statistic	4.765
Asymptotic Sig.(2-sided test)	.000

Table S5. Independent-Samples Mann-Whitney U Test of AML_dep_max across Common_Essential

Total N	17634
Mann-Whitney U	32494335.000
Wilcoxon W	34753210.000
Test Statistic	32494335.000
Standard Error	220073.064
Standardized Test Statistic	72.776
Asymptotic Sig.(2-sided test)	.000

Table S6. Independent-Samples Mann-Whitney U Test of AML_dep_max across FDA_inhibitor

Total N	17634
Mann-Whitney U	8180727.500
Wilcoxon W	8699398.500
Test Statistic	8180727.500
Standard Error	157664.042
Standardized Test Statistic	-1.756
Asymptotic Sig.(2-sided test)	.079

Table S7. Independent-Samples Mann-Whitney U Test of AML_dep_max across Cancer_inhibitor

Total N	17634
Mann-Whitney U	1230054.500
Wilcoxon W	1239924.500
Test Statistic	1230054.500
Standard Error	59993.487
Standardized Test Statistic	.091
Asymptotic Sig.(2-sided test)	.927

Table S8. Independent-Samples Mann-Whitney U Test of AML_dep_max across AML_inhibitor

Total N	17634
Mann-Whitney U	691368.000
Wilcoxon W	692964.000
Test Statistic	691368.000
Standard Error	38034.198
Standardized Test Statistic	5.237
Asymptotic Sig.(2-sided test)	.000

Table S9a. Contingency table of FDA_inhibitor * Common_Essential

			Common_Essential		
			0	1	Total
FDA_inhibitor	0	Count	14595a	2021a	16616
		Expected Count	14613.7	2002.3	16616.0
		% within FDA_inhibitor	87.8%	12.2%	100.0%
		% within Common_Essential	94.1%	95.1%	94.2%
		% of Total	82.8%	11.5%	94.2%
	1	Count	914a	104a	1018
		Expected Count	895.3	122.7	1018.0
		% within FDA_inhibitor	89.8%	10.2%	100.0%
		% within Common_Essential	5.9%	4.9%	5.8%
		% of Total	5.2%	0.6%	5.8%
Total		Count	15509	2125	17634
		Expected Count	15509.0	2125.0	17634.0

	% within FDA_inhibitor	87.9%	12.1%	100.0%
	% within Common_Essential	100.0%	100.0%	100.0%
	% of Total	87.9%	12.1%	100.0%

Each subscript letter denotes a subset of Common_Essential categories whose column proportions do not differ significantly from each other at the .05 level.

Table S9b. Chi-Squared analysis of FDA_inhibitor * Common_Essential

	Value	df	Asymptotic Significance (2-sided)	Exact Sig. (2-sided)	Exact Sig. (1-sided)
Pearson Chi-Square	3.430[a]	1	.064		
Continuity Correction[b]	3.249	1	.071		
Likelihood Ratio	3.584	1	.058		
Fisher's Exact Test				.069	.036
Linear-by-Linear Association	3.430	1	.064		
N of Valid Cases	17634				

a. 0 cells (0.0%) have expected count less than 5. The minimum expected count is 122.67.
b. Computed only for a 2x2 table

Table S10a. Contingency table of Cancer_inhibitor * Common_Essential

			Common_Essential 0	Common_Essential 1	Total
Cancer_inhibitor	0	Count	15387$_a$	2107$_a$	17494
		Expected Count	15385.9	2108.1	17494.0
		% within Cancer_inhibitor	88.0%	12.0%	100.0%
		% within Common_Essential	99.2%	99.2%	99.2%
		% of Total	87.3%	11.9%	99.2%
	1	Count	122$_a$	18$_a$	140
		Expected Count	123.1	16.9	140.0
		% within Cancer_inhibitor	87.1%	12.9%	100.0%
		% within Common_Essential	0.8%	0.8%	0.8%
		% of Total	0.7%	0.1%	0.8%
Total		Count	15509	2125	17634
		Expected Count	15509.0	2125.0	17634.0
		% within Cancer_inhibitor	87.9%	12.1%	100.0%
		% within Common_Essential	100.0%	100.0%	100.0%
		% of Total	87.9%	12.1%	100.0%

Each subscript letter denotes a subset of Common_Essential categories whose column proportions do not differ significantly from each other at the .05 level.

Table S10b. Chi-Squared analysis of Cancer_inhibitor * Common_Essential

	Value	df	Asymptotic Significance (2-sided)	Exact Sig. (2-sided)	Exact Sig. (1-sided)
Pearson Chi-Square	.087[a]	1	.769		
Continuity Correction[b]	.027	1	.870		
Likelihood Ratio	.085	1	.771		
Fisher's Exact Test				.806	.435
Linear-by-Linear Association	.087	1	.769		
N of Valid Cases	17634				

a. 0 cells (0.0%) have expected count less than 5. The minimum expected count is 16.87.
b. Computed only for a 2x2 table

Table S11a. Contingency table of AML_inhibitor * Common_Essential

			Common_Essential		Total
			0	1	
AML_inhibitor	0	Count	15470$_a$	2108$_b$	17578
		Expected Count	15459.7	2118.3	17578.0
		% within AML_inhibitor	88.0%	12.0%	100.0%
		% within Common_Essential	99.7%	99.2%	99.7%
		% of Total	87.7%	12.0%	99.7%
	1	Count	39$_a$	17$_b$	56
		Expected Count	49.3	6.7	56.0
		% within AML_inhibitor	69.6%	30.4%	100.0%
		% within Common_Essential	0.3%	0.8%	0.3%
		% of Total	0.2%	0.1%	0.3%
Total		Count	15509	2125	17634
		Expected Count	15509.0	2125.0	17634.0
		% within AML_inhibitor	87.9%	12.1%	100.0%
		% within Common_Essential	100.0%	100.0%	100.0%
		% of Total	87.9%	12.1%	100.0%

Each subscript letter denotes a subset of Common_Essential categories whose column proportions do not differ significantly from each other at the .05 level.

Table S11b. Chi-Squared analysis of AML_inhibitor * Common_Essential

	Value	df	Asymptotic Significance (2-sided)	Exact Sig. (2-sided)	Exact Sig. (1-sided)
Pearson Chi-Square	17.764[a]	1	.000		
Continuity Correction[b]	16.074	1	.000		
Likelihood Ratio	13.266	1	.000		
Fisher's Exact Test				.000	.000
Linear-by-Linear Association	17.763	1	.000		
N of Valid Cases	17634				

a. 0 cells (0.0%) have expected count less than 5. The minimum expected count is 6.75.
b. Computed only for a 2x2 table
